# Listeners do not tune in to more rewarding voices

**DOI:** 10.3758/s13414-026-03276-x

**Published:** 2026-05-15

**Authors:** Sung-Joo Lim, Lingyu Zi

**Affiliations:** Department of Psychology, Binghamton University, 4400 Vestal Parkway E, Binghamton, NY 13902 USA

**Keywords:** Speech comprehension, Attention, Reward, Speech perception

## Abstract

During conversations in the presence of other competing talkers, multiple speech streams compete for listeners’ attentional focus. Listeners must segregate speech streams, and selectively attend to the target talker while filtering out irrelevant speech. Research on value-driven attention suggests that perceptual and attentional processes are biased by prior rewarding experiences with stimuli—that is, high-valued stimuli win attentional competition. However, it remains unknown whether such effects generalize to speech perception in multitalker environments. Here, the present study investigated whether listeners can better understand speech spoken by talkers associated with higher versus lower values in challenging listening conditions. In three experiments, we used reward-based training paradigms to either explicitly or implicitly induce listeners to associate talkers’ voices with varying magnitudes of rewards. Subsequently, listeners performed a speech-on-speech intelligibility task in which the exposed voices were either the target or distractor. We found that explicit talker–reward learning did not influence speech intelligibility. In contrast, we observed that implicitly acquired talker–reward associations improved the intelligibility of high-reward talkers, but only in the most adverse listening condition. However, this effect did not replicate when we further imposed greater attentional demands by presenting target and masker talkers’ speech without spatial separation. Instead, listeners relied primarily on acoustic cues other than reward-associated talkers’ voices. Furthermore, the reward values associated with the distractor talker had no effect on interference. These findings suggest that value associated with talkers’ voices do not reliably bias listeners’ attention to overcome the complex, acoustic constraints imposed in speech-on-speech perception.

## Introduction

“Her voice is full of money.” – F. Scott Fitzgerald, *The Great Gatsby.*

In everyday life, we often encounter complex listening situations with multiple simultaneous sound sources. This cocktail-party situation (Cherry, 1953), in which multiple speakers’ speech streams compete for listeners’ attention, poses challenges to listeners’ perceptual and attentional processes. Listeners must form and segregate discrete talkers’ speech streams, selectively attend to the speech spoken by the talker of listener’s interest, and filter out other irrelevant sounds (Shinn-Cunningham, 2008). Given that speech conveys both content and the identity of the speaker (Abercrombie, 1967), the distinctive characteristics of a talker’s voice play a crucial role in identifying and processing speech amidst other competing talkers. Thus, speech intelligibility in multitalker environments fundamentally reflects the outcome of competition between auditory objects under attentional constraints.

Previous studies have highlighted that speech spoken by familiar talkers is more intelligible than that by unfamiliar talkers in cocktail-party-like situations. For example, listeners are better at comprehending speech spoken by their spouses or close friends than by strangers (Holmes et al., 2018; Johnsrude et al., 2013; Souza et al., 2013). Even short-term exposure to talkers’ voices in laboratory settings can improve listeners’ ability to selectively attend to and/or understand the speech of those talkers (Case et al., 2018; Holmes et al., 2021; Levi et al., 2011; Magnuson et al., 2021; Nygaard & Pisoni, 1998; Nygaard et al., 1994; Kreitewolf et al., 2017; Yonan & Sommers, 2000; cf. Newman & Evers, 2007). Recent work suggests this familiarity benefit is most pronounced when listeners must parse speech in the presence of competing speech streams that requires the formation, segregation, and protection of a target auditory object (Holmes & Johnsrude, 2020, 2021).

These findings raise the question as to whether familiarity is the only driver of the intelligibility advantage of familiar talkers. It remains unknown whether the affective associations or behavioral significance of familiar talkers also influence their speech intelligibility. Specifically, some familiar talkers may be more important and valuable to listeners than others. Such distinct associations can emerge through the experiences and interactions listeners have with these talkers beyond the degree of familiarity. It therefore remains unclear whether the behavioral importance or learned value associated with a talker can independently shape speech intelligibility, particularly under conditions that demand selective attention.

The value-driven attention framework (see Anderson, 2016a, 2019; Awh et al., 2012; Theeuwes, 2018, for reviews) posits that prior experience with rewards can bias attentional prioritization, such that stimuli associated with rewarding outcomes are preferentially processed even in the absence of explicit awareness. Dopaminergic reward signals play a crucial role in inducing changes in the brain’s responses, leading to preferential representation of sensory stimuli associated with rewards (e.g., Bao et al., 2001, 2004; Hui et al., 2009; Recanzone et al., 1993; Robinson & Berridge, 1993; Schultz et al., 1997). This representational enhancement is closely linked to the degree of learned importance (Rutkowski & Weinberger, 2005). The effects of reward can modulate early visual (Cheng et al., 2021; Seitz et al., 2009; Serences, 2008; Stănişor et al., 2013) as well as auditory processing (Brosch et al., 2011; David et al., 2012).

Consequently, reward history exerts a powerful influence on selective attention. Reward facilitates goal-directed attention toward task-relevant stimuli, biasing attention to prioritize stimuli that were previously associated with rewards (e.g., Awh et al., 2012; Chelazzi et al., 2013; Failing & Theeuwes, 2018). Moreover, extensive studies in the visual domain have demonstrated that reward-associated stimuli can also “automatically” capture attention regardless of their relevance to the current task (e.g., Anderson & Yantis, 2013; Anderson et al., 2011a, 2011b; MacLean & Giesbrecht, 2015; MacLean et al., 2016). Thus, even without physically salient features, rewards can shape the “learned saliency” of a stimulus. This suggests that reward learning (thus, reward history) plays a crucial role in determining which stimuli or objects win the competition for limited attentional resources (Anderson et al., 2011a; Awh et al., 2012; Chelazzi et al., 2014).

Based on the framework of value-driven attention, it is conceivable that listeners may be more effective in processing speech from talkers deemed to be of high behavioral importance, as they can preferentially parse valuable speech features associated with rewards, especially in situations that demand focused attention. In other words, if voices function as auditory objects competing for representation, reward history associated with a talker could, in principle, alter the outcome of that competition. Under this account, reward might enhance the effective salience of a talker’s voice, facilitating stream formation and increasing resistance to informational masking. However, empirical evidence in the auditory and speech domains remains mixed.

Within the context of phonetic recalibration, monetary rewards have been shown to bias listeners’ post-perceptual decisions when rewards are tied to specific responses (Connine & Clifton, 1987). However, when rewards are associated with particular talkers’ speech, listeners do not show enhanced perceptual learning of talker-specific idiosyncratic pronunciations for high-rewarded versus low-rewarded talkers, irrespective of explicit awareness of reward contingencies (Mechtenberg et al., 2025). Based on these two studies, it appears that when reward can influence speech categorization, its effects have been attributed primarily to decision-level biases rather than changes in perceptual representations. However, these studies did not exert demands for selective attention (i.e., no distractor speech or other sounds) on the listeners.

In contrast, other auditory studies have demonstrated value-driven attentional effects, particularly under conditions involving competing stimuli. When different reward magnitudes are associated with specific words, high-reward stimuli can interfere with goal-directed attention to other task-relevant targets, even across modalities (e.g., Anderson, 2016b; Kim et al., 2021). Similarly, Asutay and Västfjäll (2016) showed that nonspeech tones previously associated with high reward impaired auditory target detection performance when presented as distractors in a dual-stream auditory task, whereas low-reward distractors were more effectively suppressed. Notably, in these studies, reward associations were tied to relatively discrete and concrete auditory stimuli.

Taken together, the current literature suggests two open questions. First, it remains unclear whether the value-driven attentional modulation observed with discrete auditory stimuli (e.g., individual words or discriminable frequency tones) generalize to more complex auditory objects such as talkers’ voices. Second, it is unknown whether the impact of reward is more notable when listeners’ attentional resources are constrained; in other words, whether reward history modulates speech intelligibility specifically in parsing speech in multitalker settings, where stream segregation and selective attention are essential.

To address these questions, this study examined whether prior rewarding experiences with talkers’ voices could differentially impact the intelligibility of their speech in challenging listening environments involving competing speech. Especially, if reward can boost the salience of associated voices and biases attentional selection, we expected the effect of rewards should be most evident under perceptually demanding conditions, such as when target audibility is reduced and informational masking is high. Furthermore, prior work suggests that explicit awareness of reward contingencies is not necessary for value-driven attentional effects (e.g., Anderson et al., 2011a, 2011b; Asutay & Västfjäll, 2016). We therefore tested whether explicit versus incidental learning of talker–reward associations differentially influences subsequent speech intelligibility.

To this end, we had participants engage in a training task that provided trial-by-trial feedback with rewards while they listened to speech spoken by four female talkers over 2 days. Here, we varied the magnitude of rewards (high values vs. low values) associated with each talker while ensuring equal exposure across all talkers, thereby equating familiarity. Following exposure, participants identified words from a sentence spoken by a target talker while attempting to ignore another competing sentence, simultaneously spoken by a masker talker. We evaluated the effect of talker–reward associations on speech intelligibility when listeners focused on (or ignored) speech spoken by the high-valued versus low-valued talkers.

Furthermore, to investigate whether explicit awareness of talker–reward associations was necessary for speech intelligibility benefits, we employed two distinct reward-learning paradigms that differed in how listeners acquired the talker–reward associations during the exposure phase. In one task, participants performed a talker identification task, in which they identified talkers based on their voices and received trial-by-trial feedback with specific reward values; therefore, task-relevant dimensions were explicitly linked to receiving high or low rewards. In the other task, participants were incidentally exposed to talker–reward associations while performing a 2-back auditory working memory task, in which listeners judged whether the speech content was the same as that from two trials ago; hence, task demands were orthogonal to talkers or rewards.

We hypothesized that if reward facilitates top-down attention to task-relevant speech features (e.g., Hickey et al., 2010; Raymond & O’Brien, 2009; Serences, 2008), then listeners would exhibit enhanced processing of target speech when it is produced by high-reward talkers compared with low-reward talkers. We also hypothesized that if reward exerts an influence that automatically captures bottom-up attention to rewarding stimuli (Anderson et al., 2011a, 2011b; Failing & Theeuwes, 2014), then listeners would have greater difficulty processing the target speech when they must ignore the masking speech from a high-value talker. Additionally, if reward-driven attentional modulation is a result of conscious awareness of reward contingencies, then the effects on speech intelligibility should be stronger after explicit training to associate talkers’ voices with rewards. Alternatively, if incidental/implicit reward learning is particularly effective for acquiring complex, speech-like auditory categories (Chandrasekaran et al., 2014; Lim & Holt, 2011; Lim et al., 2014; Seitz et al., 2010; Vlahou et al., 2012) and that explicit awareness is not necessary for value-driven attentional bias (e.g., Anderson et al., 2011a, 2011b), then incidental exposure to talker–reward contingencies should be sufficient to yield speech intelligibility benefits. Evaluating these possibilities allows us to determine whether reward history meaningfully alters auditory object competition in multitalker speech perception.

## Experiment 1

### Methods

#### Participants

No prior study has evaluated the effect of voice–reward associations on speech intelligibility. Thus, to determine the appropriate a priori sample size, we consulted two studies of analogous paradigms. The first, which examined the effect of reward on auditory attentional bias using simple nonspeech tones, reported a large effect (*f* = 0.83) with 16 participants (Asutay & Västfjäll, 2016). The second, which examined the interaction between voice familiarity and the degree of adverse listening on speech intelligibility, reported a medium effect (*f* = 0.27; Johnsrude et al., 2013). Our a priori power analysis using G*Power 3.1 (Faul et al., 2009) with *α* =.05 and 1 − *β* =.80 based on the medium effect (*f* = 0.27) yielded a target sample size of 21. Assuming attrition across a two-day experiment, we recruited 26 participants. Of these, one did not return for the second session. The remaining 25 native speakers of American English (15 women, nine men, one other; mean age = 18.5 ± 0.65, range: 18–20 years) participated in the study. All participants were recruited from Binghamton University and received course credit for their participation. All participants had self-reported normal hearing and normal or corrected-to-normal vision. None reported having any speech or language disorder. This study was approved by the Institutional Review Board of Binghamton University. Prior to their participation, all individuals provided written informed consent.

### Stimuli

The Boston University Gerald (BUG) corpus (Kidd et al., 2008) was used in the study. The corpus contains five categories of words that can be concatenated for presentation as sentences in the form of “[Name] [verb] [number] [adjective] [noun].” An example sentence from the BUG corpus is “Sam sold three old socks.” For each word category, there were eight distinct monosyllabic words, comprising a closed set (Table 1). In this study, we used the recordings of four out of 10 female speakers from the BUG corpus; the voices of the chosen speakers were independently rated as distinct by four naïve native American-English listeners.

**Table 1:** The 40-word Boston University Gerald (BUG) Corpus closed-set word choices (Kidd et al., 2008)

Name	Verb	Number	Adjective	Noun
Bob	bought	two	big	bags
Jane	found	three	cheap	cards
Jill	gave	four	green	gloves
Lynn	held	five	hot	hats
Mike	lost	six	new	pens
Pat	saw	eight	old	shoes
Sam	sold	nine	red	socks
Sue	took	ten	small	toys

The words were arranged in eight rows and five columns, where each column is a word category (name, verb, number, adjective, and noun) and a choice of one word from each column in order from left to right produces a syntactically correct but unpredictable sentence

During the talker–reward contingency exposure phase, participants only heard spoken digits from the number category of the corpus. During the speech intelligibility test phase, participants heard the full five-word, matrix sentences made from concatenating one randomly selected word from each category.

#### Task design and procedure

The experiment was composed of two parts: an exposure phase and a test phase. Both phases were completed in sound-attenuated booths. All auditory stimuli were delivered using Sennheiser HD-280 pro headphones. The experiment was controlled via Psychtoolbox-3 (Brainard, 1997) and MATLAB (MathWorks, Inc., Natick, MA).

##### Talker–reward exposure phase

**Fig. 1:**
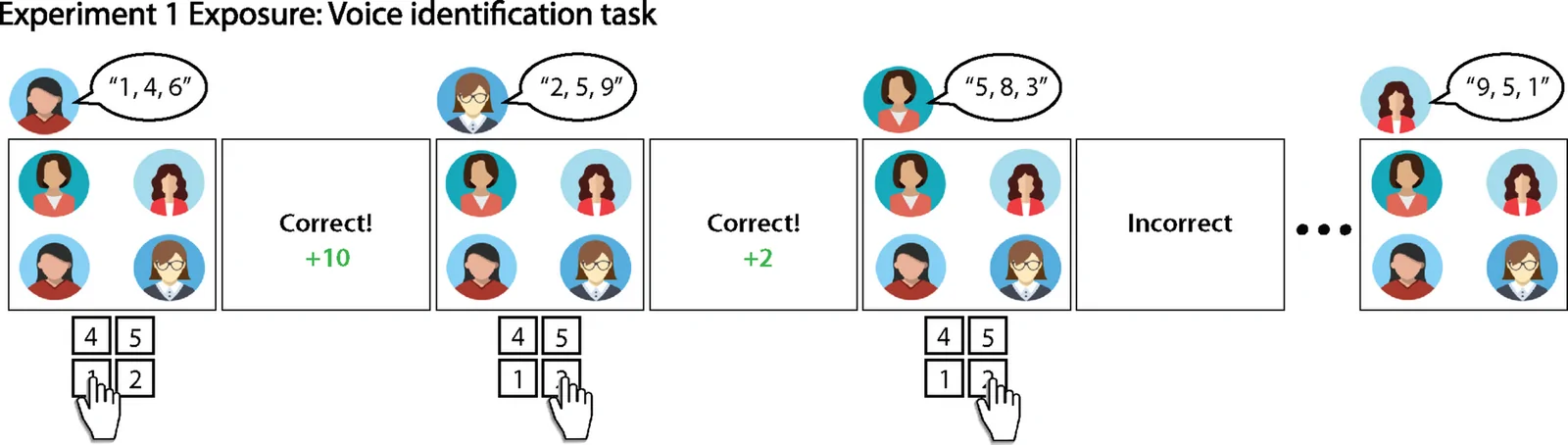
Experiment 1 exposure task design. Participants were exposed to the talker–reward associations through a talker identification task. On each trial, participants heard a series of three digits spoken by one talker (out of four talkers) and were asked to identify the talker (shown as avatars) using the corresponding key on a number pad. After each response, participants received trial-by-trial feedback. If participants responded correctly, the feedback screen presented the reward values associated with the talker (80% high-reward and 20% low-reward points for high-valued talkers; 20% high-reward and 80% low-reward points for low-valued talkers). Participants also received a total amount of points accrued on the feedback screen (not depicted here). (Color figure online)

First, participants performed a talker identification task, through which they were exposed to four talkers’ voices and the corresponding reward associations (Fig. 1). On each trial, participants heard a sequence of three randomly selected digits spoken by one talker and asked to identify the talker by matching an avatar out of four presented on the screen by pressing the corresponding number key on a keypad within a 2.5-s response time window. The three digits were presented with no temporal gaps (i.e., 0-s interstimulus interval). The stimulus duration naturally varied with distinct talkers’ speaking styles. On average, the stimulus duration was (*M* ± *SD*) 1.19 ± 0.17 s. Participants received visual feedback on each trial indicating the correctness of the response. In addition, for each correct response, the feedback screen displayed the reward value of that trial (i.e., the number of task points). Based on prior studies showing the effectiveness of immediate feedback in perceptual category learning compared with the delayed feedback (Chandrasekaran et al., 2014; Maddox et al., 2003; Seitz & Watanabe, 2005), the current task provided feedback immediately after each response, and it was displayed for 1.5 s on the screen.

Two of the four talkers were assigned to be high-valued talkers, and the remaining two were assigned to be low-valued talkers. Following Anderson et al. (2011a, 2011b), reward associations followed probabilistic distributions. Correct identification of the high-valued talkers led to high-reward feedback (10 points) on 80% of trials and low-reward (2 points) on the remaining 20%. These probabilities of high- versus low-reward feedback were reversed for correct identifications of the low-valued talkers (such that they earned 2 points on 80% and 10 points on 20% of trials). The feedback screen also displayed the total accumulated reward points. It is of note that the points gained during exposure were task-internal and did not translate into monetary rewards. The associations between the magnitudes of reward values (high vs. low) and talkers were counterbalanced across participants.

Listeners heard a total of four female talkers in the course of the experiment. Participants went through a total of 16 blocks of 80 trials each (i.e., 20 trials presented the spoken digits of each talker for each block). Participants were asked to respond as quickly and as accurately as possible. Participants were told they should obtain as many reward points as they could during the task. Each stimulus was presented an equal number of times throughout the training (i.e., 40 repetitions per stimulus).

The total number of trials presented during exposure here (i.e., 1,280 trials) was followed based on the Anderson et al. (2011a, 2011b) that demonstrated robust value-driven attentional capture with visual features. We provided the exposure task over the two sessions in order to reduce participant fatigue and to potentially enhance training effect via memory consolidation during sleep (Earle & Myers, 2015; Fenn et al., 2003; Tamaki et al., 2020).

##### Speech intelligibility test phase

**Fig. 2:**
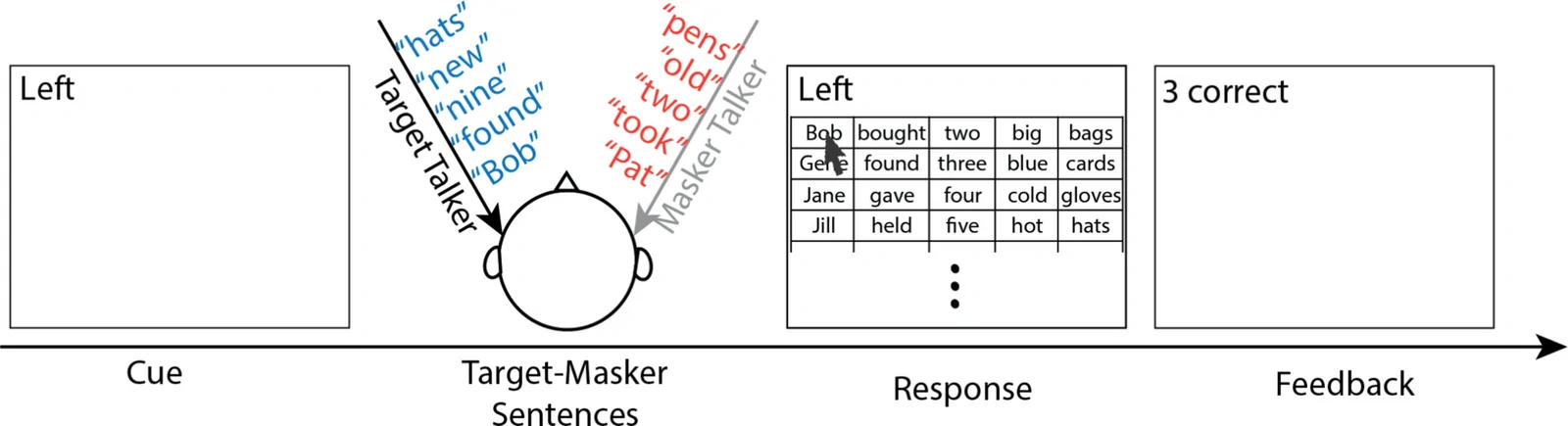
Trial structure of the speech intelligibility test following the exposure phase in both Experiments 1 and 2. In each trial, participants received a cue (0.5 s) indicating the direction of the target speech (left vs. right) on the screen. Two sentences (a target and a masker) were presented simultaneously through headphones and sentences with approximately 30 degrees separation on the lateral positions equally away from the center. After participants heard the sentences, the button boxes appeared on the screen and they selected each word from the target sentence. At the end of the trial, participants were informed as to the number of words that they correctly recalled without any reward points. The direction of the target sentence was randomized across trials in each block. The colors of the sentences indicate distinct talkers. (Color figure online)

Upon the completion of the exposure task, participants performed a speech-intelligibility test (Fig. 2). On each trial, participants heard two simultaneously presented BUG sentences (Table 1), each spoken by a different talker drawn from the exposure phase. The two sentences were spatially separated from each other by manipulating the interaural time difference of the stimuli by ± 132 μs (roughly 15° left and right of center based on the Woodworth’s model). The purpose of this manipulation was to enable listeners to reduce masking effects caused by same-gender voices (Kidd et al., 2016; Oh et al., 2021). Prior to hearing the sentences, participants were instructed to listen to the sentence spoken by a target talker, indicated by a directional cue (left vs. right) shown on the screen. After hearing the sentences, an array of the corpus words was displayed on the screen, and participants were instructed to select the five words in the target sentence using a computer mouse. Feedback was provided on each trial indicating the number of words correctly identified from the target sentence, but no reward points were provided. Words in the target and masker sentences were never the same, and no same words were presented in adjacent trials.

Four conditions varied in the reward values associated with the target and masker talkers: 2 (target-talker values: high vs. low reward) × 2 (masker-talker values: high vs. low reward). Furthermore, we assessed these conditions across the four different levels of target-to-masker ratios (TMRs; −20, −15, −10, and −5 dBs); this was done by adjusting the amplitude of the target sentences while keeping the masker sentences at a constant level of 70 dB sound pressure level (SPL).

Each participant completed a total of 256 test trials, organized in eight blocks of 32 trials. Within each block, each target and masker talker pair condition was equally tested, and the direction of target versus masker talker was balanced. The four levels of TMR conditions varied across blocks; all trials in a block had the same TMR level, and no two consecutive blocks tested the same TMR level. The order of blocks was counterbalanced across participants using Latin-square permutation. We ensured that all speech tokens of each talker were presented an equal number of times across the conditions.

Participants completed the experiment across two 1.5-h sessions. In the first session, participants performed 960 trials of the talker–reward exposure task; in the subsequent session, participants completed the remaining 320 trials of the exposure task prior to performing the speech intelligibility test. Participants completed the second session within 2 days after completing the first.

#### Data analysis

We analyzed participants’ performances in both exposure and test phases of the experiment. For the exposure phase (i.e., the voice identification task), participants’ single-trial performance accuracy and reaction times (RTs) were analyzed using (generalized) linear mixed-effects models. Prior to the analysis of RTs, the data were log-transformed to approximate normality. In addition, we excluded RTs of incorrect and outlier trials, on which participants’ log-transformed RTs were greater than 2 standard deviations from each participant’s mean. The models included a fixed factor of *talker–reward value* condition (high vs. low) and by-participants and by-talker random intercepts.

To analyze the performances on the post-exposure speech-intelligibility test, we analyzed each participant’s accurate recall of each word in the target sentence on each trial. The generalized mixed-effects model included fixed factors of *target talker–reward value* (high vs. low), *masker talker–reward value* (high vs. low), and *TMRs* (−20, −15, −10, and −5 dBs). The model also included by-participants and by-stimulus-item random intercepts.

For all mixed-effects models described above, fixed factors were sum-coded. To determine the appropriate random-effects structure, we conducted a forward model comparison procedure. Starting with a model that included by-participant random intercepts, we added by-participant random slopes for fixed factors in a stepwise manner. Random slopes were retained when their inclusion improved model fit and did not result in convergence or singularity issues (Matuschek et al., 2017).

All data were analyzed with mixed-effects models implemented in R (*lme4* package). The significance of fixed effects of all models was evaluated using Type III Wald χ^2^ test (*car* package) at an α = 0.05. This approach provides an omnibus test of each factor and interaction, enhancing interpretability of predictors with multiple (> 2) levels. Any significant effects found in the mixed-effects models were followed by post hoc tests using estimated marginal means (*emmeans* in R; Lenth, 2024) and the Holm–Bonferroni method was used to correct for multiple comparisons.

### Results

#### Results of exposure phase

**Fig. 3:**
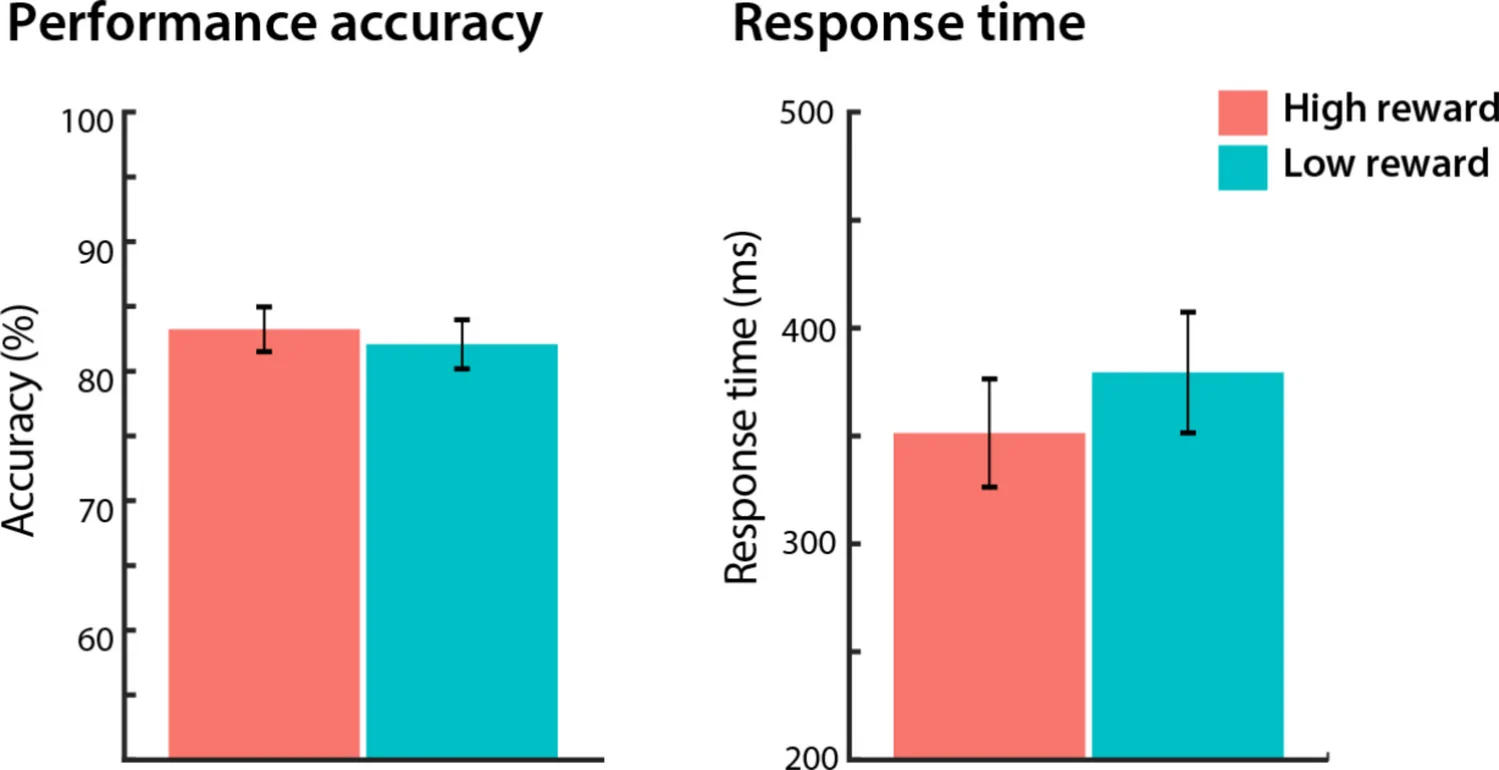
Task performance in each talker–reward value condition during the Experiment 1 exposure task (i.e., talker identification). Left, response accuracy. Right, average response time on the correct trials. The average performances across all blocks are shown. The error bars indicate ± 1 standard error of the mean (*SEM*). (Color figure online)

Listeners’ performances during the reward-based voice exposure task (i.e., voice identification) are illustrated in Fig. 3 and Table 2. We first assessed whether the different magnitudes of reward values associated with the talkers influenced listeners’ ability to identify the four female talkers during the exposure phase. Listeners were not more accurate at identifying the voices of high-valued than low-valued talkers, as shown by the linear mixed-effects model revealing no significant effect of the talker–reward condition, χ^2^(1) = 0.52, *p* = 0.47. In addition, listeners showed a slight tendency to identify high-reward talkers faster than low-reward talkers, but this difference was not statistically significant, χ^2^(1) = 2.08, *p* = 0.15.

**Table 2:** Mean (± *SD*) talker identification accuracy and response time for each talker–reward condition in Experiment 1

	Accuracy (%)	Response time (ms)
High reward	83.22 ± 8.65	351.4 ± 125.5
Low reward	82.08 ± 9.49	379.4 ± 140.0

We further examined whether participants’ identification responses reflected a response bias toward high-valued talkers, as reported by Connine and Clifton (1987). Specifically, we analyzed participants’ response patterns in the four-alternative forced-choice (4AFC) task when they heard speech from high-valued versus low-valued talkers. On average, responses corresponding to high- and low-valued talkers were 25.1% and 24.9%, respectively. A mixed-effects linear model on the response probabilities revealed no significant effect of talker-value contingency, *t*(98) = 0.68, *p* = 0.50, indicating that listeners were not biased to select responses associated with high-valued talkers over low-valued talkers.

#### Results of test phase in accurate identification of target speech

**Fig. 4:**
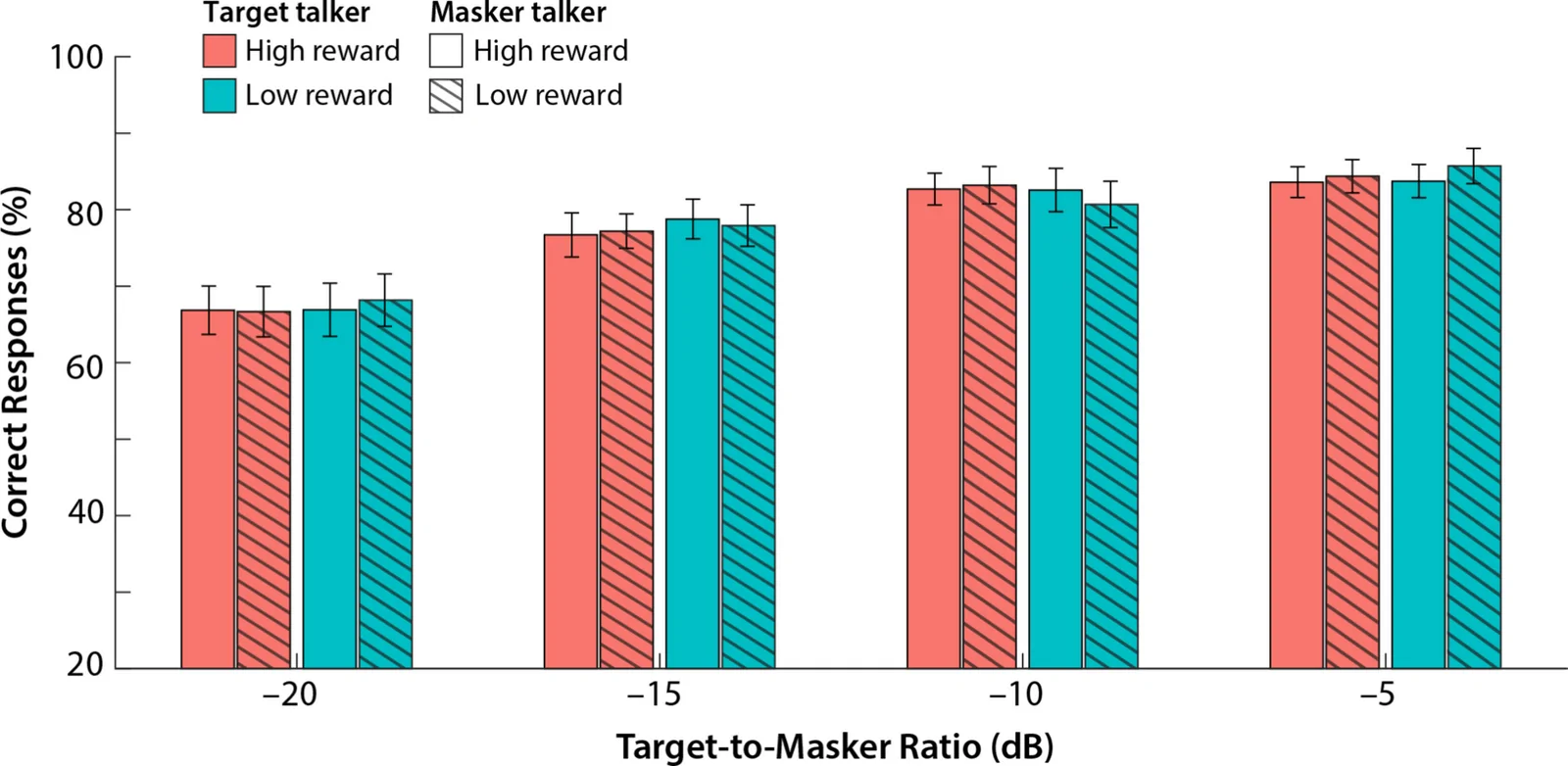
Experiment 1 participants’ performances on the speech intelligibly test for each condition varied in reward values of the target × masker talkers. The red and blue colors of the bars indicate performances when the target talker is associated with high rewards and low rewards, respectively. The open and shaded textured bars indicate the reward values associated with the masker talkers. The error bars indicate ± 1 *SEM*. (Color figure online)

Next, we assessed whether the talker–reward contingencies learned during the exposure phase affected listeners’ subsequent speech intelligibility across the four levels of TMR (Fig. 4). The generalized mixed-effects model on listeners’ accuracy in identifying target words revealed a significant main effect of TMR, χ^2^(3) = 182.69, *p* < 0.0001. As expected, listeners were less accurate at identifying words from target sentences as the listening condition became more challenging (i.e., with lower TMRs). However, the same model did not reveal any significant main or interaction effects related to the reward values of the target or masker talkers (all *p* values > 0.19; see Table 3).

**Table 3:** Mixed-effects logistic modeling results on the speech intelligibility test performance in Experiment 1

	χ^2^	*df*	*p*
TMR	182.69	3	< 0.0001*
Target talker value	0.79	1	0.37
Masker talker value	0.48	1	0.49
TMR × target talker value	4.49	3	0.21
TMR × masker talker value	4.37	3	0.22
Target talker × masker talker value	0.27	1	0.60
TMR × target talker × masker talker value	4.82	3	0.19

*TMR *target-to-masker ratio. * denotes the significant effect based on Type III Wald χ^2^ test

#### Results of test phase in masker confusion

We further analyzed whether the extent of distractibility from masker speech depended on the talker–reward contingencies. We estimated the proportion of confusion errors where listeners reported words from masker sentences out of the total number of error responses across all conditions. A separate linear mixed-effects model was used to examine the effects of the conditions on the proportion of confusion errors. As expected, the model revealed a significant main effect of TMR, χ^2^(3) = 39.55, *p* < 0.0001, such that masker confusion error rate increased with lower TMR (average masker error rates: *M*_–20 dB_ = 4.34 ± 1.54%, *M*_–15 dB_ = 3.16 ± 1.18%, *M*_–10 dB_ = 2.52 ± 0.74%, *M*_–5 dB_ = 2.67 ± 1.03%). However, there were no significant effects related to the reward values associated with the target or masker talkers (all χ^2^ values < 4.19; *p* values > 0.24).

### Discussion

Experiment 1 results indicate that talkers’ voices, previously associated with varying magnitudes of reward through a voice identification task, did not impact listeners’ ability to track the speech spoken by these talkers in adverse listening conditions. Specifically, participants did not process speech from high-valued talkers (i.e., target talkers) more accurately, nor did they have more difficulty ignoring speech spoken by high-valued talkers (i.e., masker talkers).

One potential explanation for our findings is that since listeners had equal amounts of exposure to all four talkers, they acquired equal levels of familiarity with the voices (e.g., Holmes et al., 2021) regardless of the varying reward values associated with the talkers. However, another possibility is that the rewards provided during voice identification training may have led listeners to focus on voice features for identifying “who the talker is” rather than on speech-specific features. Although acoustic features useful for identifying talkers and comprehending speech are intertwined (e.g., Lee & Perrachione, 2022; Levi, 2019; Perrachione et al., 2011; Perrachione & Wong, 2007), listeners may have perceptually weighted more on the talker-identifying features, such as average fundamental frequency (F0), vocal tract lengths, formant dispersion, that are generally universal in characterizing vocal qualities of talkers independent of linguistic factors (Perrachione et al., 2019). Because the present study did not systematically examine which acoustic features listeners used to distinguish talker identity, we cannot determine which specific features were prioritized during training. Nevertheless, listeners’ ability to explicitly recognize talkers’ voices may be dissociable from their speech intelligibility (Holmes et al., 2018, 2021). This suggests that listeners may have directed their attention toward voice-identifying features that do not necessarily facilitate speech processing, potentially explaining why prior exposure to talker–reward associations did not generalize to the intelligibility test. .

Another relevant consideration is the nature of reward learning. Previous reward-based perceptual learning studies have emphasized the effective role of implicit/incidental exposure to stimulus–reward pairing compared with explicit tasks. According to the task-irrelevant perceptual learning framework (Seitz & Watanabe, 2005, 2009), explicit top-down attention to features deemed relevant to receiving rewards could constrain perceptual learning (Gutnisky et al., 2009) because learners selectively attend to reward-relevant features while filtering out other task-irrelevant features. In contrast, implicit or incidental exposure to reward–stimulus associations may promote perceptual learning in a more stimulus-driven manner, as reward can enhance processing of all concurrent stimulus features irrespective of their task relevance. To examine this possibility, we conducted a follow-up experiment to implicitly expose listeners to the reward–talker contingencies while their attention is directed to speech (i.e., word-level) information.

## Experiment 2

The main purpose of Experiment 2 was to examine whether incidental exposure of talker–reward associations influenced listeners’ ability to process speech. Prior to the speech intelligibility test, Experiment 2 participants had equal amounts of exposure to the four talkers’ voices as in Experiment 1. However, the critical difference was that participants were incidentally exposed to talker–reward contingencies while performing an auditory 2-back working memory task based on spoken words. In this task, listeners received different magnitudes of rewards associated with talkers after each correct response; however, the talkers’ voices and the reward contingencies were orthogonal to the task demands. The other difference in the exposure task was that participants heard each talker’s speech in separate blocks in order to prevent the additional processing costs associated with trial-by-trial talker variability, which has been extensively documented in the literature (e.g., Choi et al., 2018; Lim et al., 2019, 2021; Magnuson & Nusbaum, 2007; Nusbaum & Morin, 1992; Wong et al., 2004; see Luthra, 2024 for a review).

### Methods

#### Participants

We used the same criteria as in Experiment 1. A new sample of 22 Binghamton University undergraduates (20 women, two men; mean age: 18.6 ± 0.60, 18–20 years) participated this experiment and received course credit. All participants met the same inclusionary/exclusionary criteria as in Experiment 1, and all provided written informed consent. Six additional participants were recruited for the study but were excluded from data analysis. Of these, five participants completed the first session but did not return for the second. One participant partially completed the test phase on the second session but was identified as an outlier due to low performance (i.e., below 40% on average across the completed test trials), which was more than 3 standard deviations below the group mean.

#### Stimuli, task design, and procedure

**Fig. 5:**
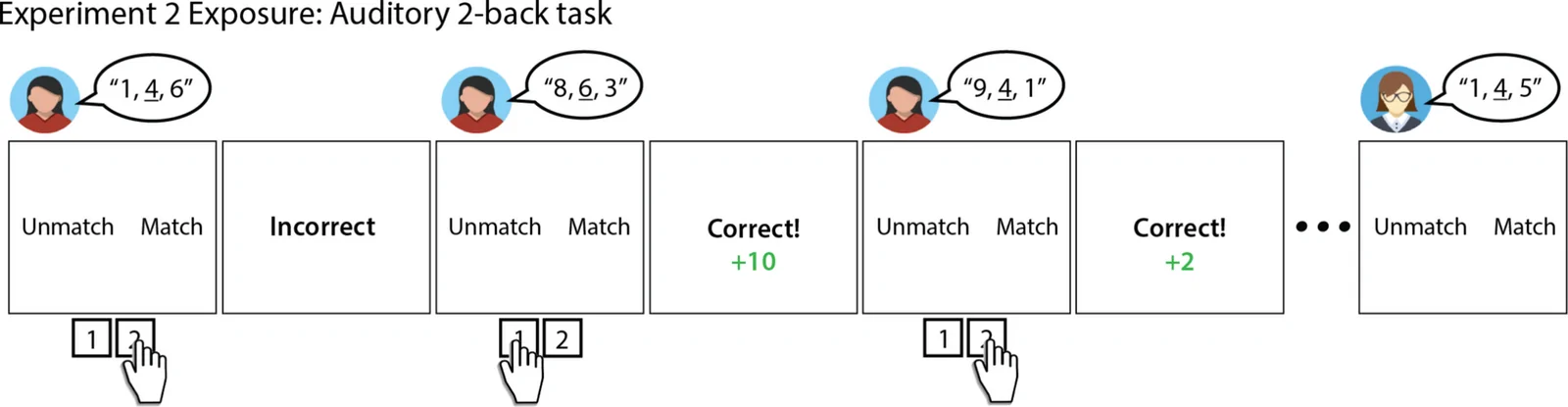
Experiment 2 exposure task. Participants performed an auditory 2-back training task to implicitly learn talkers’ voice and reward–value associations. On each trial, participants heard a sequence of three digits spoken by one talker and indicated whether the middle digit of the current trial matched (or not) the middle digit in the two trials ago. After each response, listeners received feedback showing the correctness of their responses as well as the reward value associated with the voice of the talker. The reward points received for correct responses corresponded to whether the talker is a high-value or a low-valued talker, as in Experiment 1. The reward–talker mapping was counterbalanced across participants. (Color figure online)

Except for the task design of the exposure phase, the sound stimuli, speech intelligibility test, and study procedures were identical to Experiment 1. As illustrated in Fig. 5, participants heard a sequence of three digits presented in each trial and performed an auditory 2-back working memory task. Participants indicated whether the middle digit of the sequence of the current trial matched that from two trials ago. They pressed the corresponding number keys on each trial within a 2.5-s response time window. On each block, 30% of trials were match trials. Within each block, participants heard a single, fixed talker to avoid the additional processing costs associated with trial-by-trial talker variability (Magnuson & Nusbaum, 2007; Nusbaum & Morin, 1992).

After each correct response in the 2-back task, participants were presented with a feedback screen indicating the number of reward points earned on the trial. Much as in Experiment 1, correct responses to words spoken by two talkers resulted in high-value rewards (10 points) on 80% of trials and low-value rewards (2 points) on 20% of trials, and the probabilities were reversed for the speech of the other two low-valued talkers. The task-relevant dimension (speech content) was orthogonal to the dimension contingent on reward values (talkers’ voices).

As in Experiment 1, Experiment 2 participants completed a total of 1,280 trials in the exposure phase task across 16 blocks. The blocks alternated between different talkers, and the order of blocks was counterbalanced across participants using Latin-square permutations. All participants completed the training across two separate sessions held within 2 days. Following the exposure phase on the second session, participants completed the speech intelligibility test, which was identical to that of Experiment 1 (Fig. 2). One participant elected to withdraw from the study after completing only 64 trials from the intelligibility test phase.

### Results

#### Results of exposure phase

**Fig. 6:**
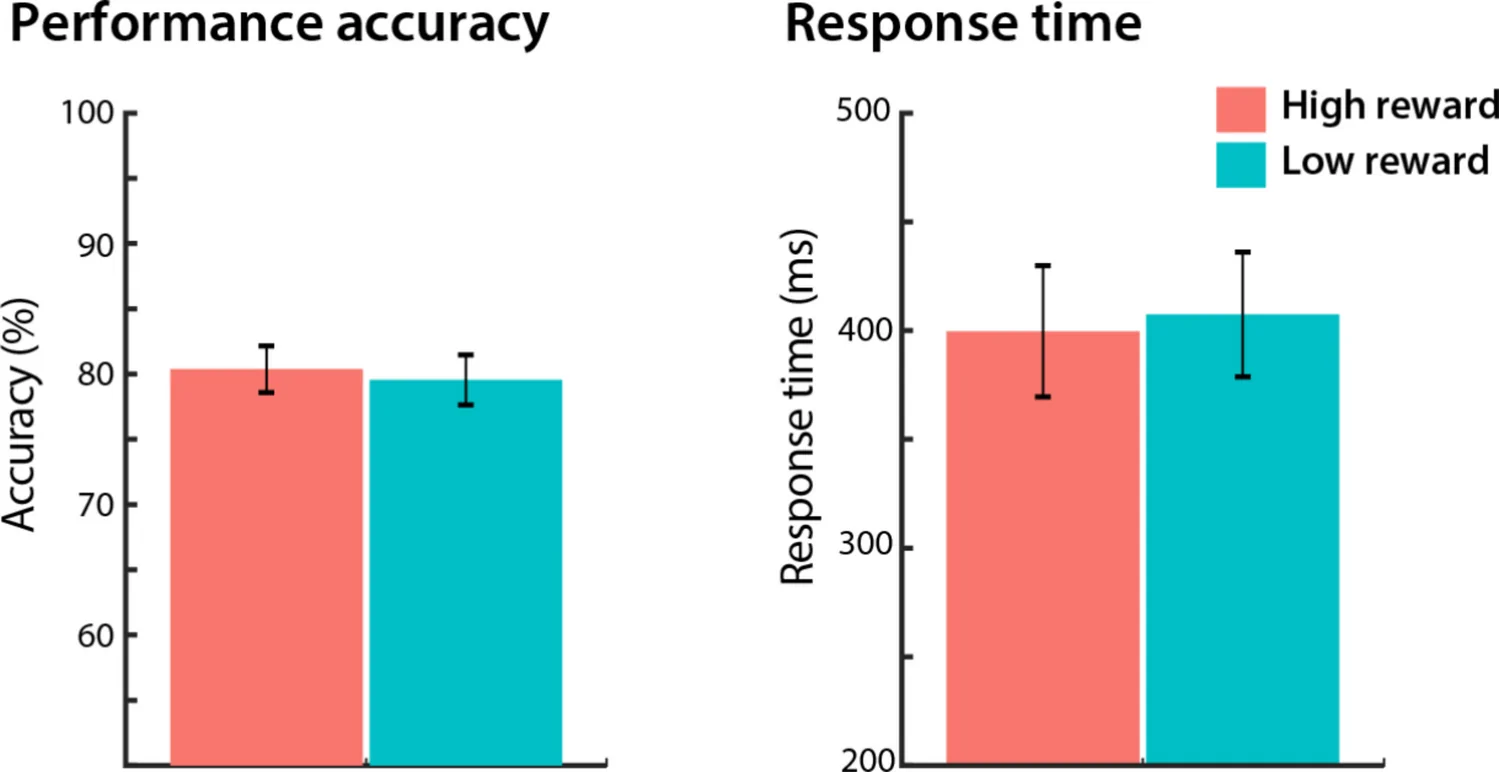
Average task performances in the auditory 2-back task (Experiment 2 exposure phase) in each talker–reward condition. Left, response accuracy. Right, average response time on the correct trials. The same illustration scheme as in Fig. 3 is used. (Color figure online)

Table 4 and Fig. 6 illustrate listeners’ performance during the exposure phase while they performed the auditory 2-back task. The mixed-effects model on listeners’ 2-back task accuracy a marginal effect of talker-reward value condition, χ^2^(1) = 2.82, *p* = 0.093. Listeners’ response times on the auditory 2-back task did not differ whether the words were spoken by a talker who had high or low reward values, χ^2^(1) = 0.13, *p* = 0.72. These nonsignificant effects of talker–reward associations during the auditory 2-back task were expected as the task responses were unrelated to the reward contingencies. The post-experiment questionnaire confirmed that none of the Experiment 2 listeners were aware of the association between the rewards and the talkers’ voices.

**Table 4:** Mean (± *SD*) accuracy and response time on performing the auditory 2-back task for each talker–reward condition in Experiment 2

	Accuracy (%)	Response time (ms)
High reward	79.62 ± 8.95	400.5 ± 138.1
Low reward	79.01 ± 9.18	407.4 ± 131.4

#### Results of test phase in correct identification of target speech

**Fig. 7:**
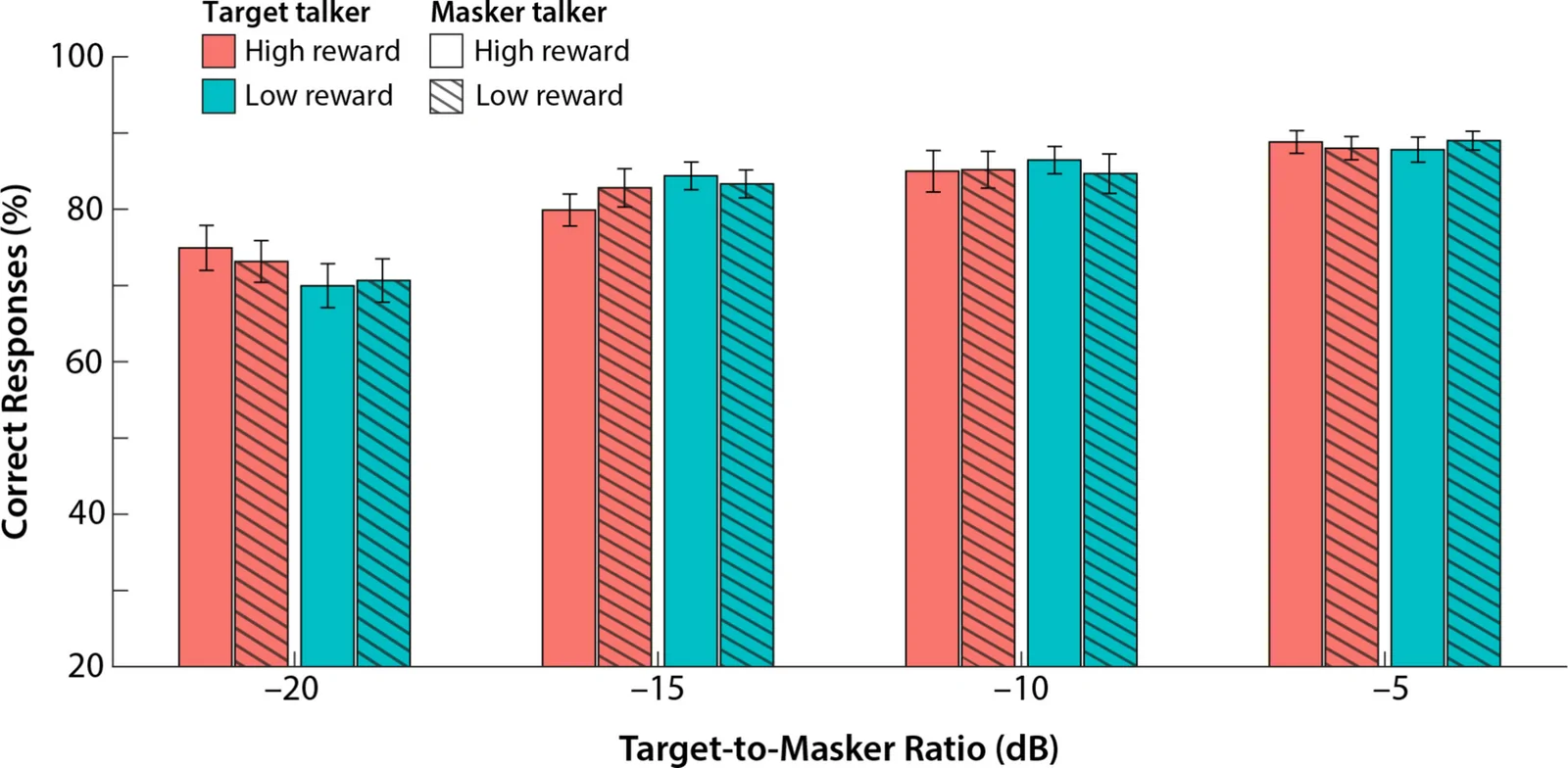
Experiment 2 participants’ performances on the speech intelligibly test for each condition varied in reward values of the target × masker talkers. The colors of the bars indicate the reward values of the target talker; the solid and shaded bars indicate the reward values of the masker talker. The error bars indicate ± 1 *SEM*. (Color figure online)

To examine whether implicitly learned talker–reward value associations can affect intelligibility of speech spoken by these talkers, we analyzed listeners’ word recall accuracy for the target sentences during the speech intelligibility test. Figure 7 shows listeners’ performances in each test condition and Table 5 lists the generalized linear mixed-effects modeling result. As expected, the model revealed a significant main effect of TMR, χ^2^(3) = 156.04, *p* < 0.0001, indicating that listeners’ overall target word recall accuracy was lower as the listening condition became more adverse.

**Table 5:** Mixed-effects logistic modeling results on the speech intelligibility test performance in Experiment 2

	χ^2^	*df*	*p*
TMR	156.04	3	< 0.0001*
Target talker value	0.23	1	0.63
Masker talker value	0.07	1	0.79
TMR × target talker value	16.66	3	0.0008*
TMR × masker talker value	0.50	3	0.92
Target talker × masker talker value	0.07	1	0.80
TMR × target talker × masker talker value	8.77	3	0.033*

*TMR* target-to-masker ratio. * denotes significant effects based on Type III Wald χ^2^ test

Moreover, the identical linear model revealed no main effect of target-talker value, χ^2^(1) = 0.23, *p* = 0.63, but there was a significant TMR × target-talker value interaction effect, χ^2^(3) = 16.66, *p* = 0.0008. Post hoc tests revealed that listeners were significantly more accurate at identifying target words when they were spoken by high-valued talkers than by low-valued talkers at the lowest TMR (at −20 dB: estimate = 0.21, *SE* = 0.059, *z* = 3.47, Holm–Bonferroni corrected *p* = 0.002). However, the reward value of the target talkers did not influence listeners’ speech intelligibility at higher TMR levels (−15 dB TMR: estimate =  −0.16, *SE* = 0.069, z =  −2.35, *p*_corrected_ = 0.057; −10 dB TMR: estimate = 0.031, *SE* = 0.075, *z* = 0.41, *p*_corrected_ = 1.00; −5 dB TMR: estimate =  −0.006, *SE* = 0.080, *z* = 0.08, *p*_corrected_ = 1.00).

The model also revealed a significant effect of TMR × target-talker value × masker-talker value interaction, χ^2^(3) = 8.77, *p* = 0.033. However, follow-up interaction contrasts in each TMR level did not remain significant after correcting for multiple comparisons (all corrected *p* values > 0.15), suggesting that there were no reliable pairwise differences at any specific TMR level.

No other significant main or interaction effects related to the value of the masker talker were observed (all *p* values ≥ 0.63). This pattern suggests that whether the masker talker was associated with high-reward or low-reward values did not affect listeners’ performance in identifying target words.

#### Results of test phase in masker confusion

To further examine the effects of the conditions on the proportion of masker confusion errors listeners made (i.e., reporting words from the masker talker), we conducted a separate linear mixed-effects model analysis. We found a significant main effect of TMR, χ^2^(3) = 17.52, *p* = 0.0006, indicating that the confusion error response rate increased as the relative amplitude of the target talkers’ speech decreased (average masker error rate; *M*_–20 dB_ = 4.71 ± 1.68%, *M*_–15 dB_ = 3.11 ± 0.77%, *M*_–10 dB_ = 2.78 ± 1.05%, *M*_–5 dB_ = 2.94 ± 1.10%). The model also revealed a significant TMR × masker-talker value interaction, χ^2^(3) = 8.37, *p* = 0.039; however, post hoc tests did not reveal any robust significant of masker-talker value in any of TMR levels after correcting for multiple comparisons (all *p* values > 0.12). No other effects related to the reward values of the target or the masker talkers were significant (all χ^2^ values < 6.87; *p* values > 0.08).

#### Post hoc* Experiment 1 versus 2 comparisons*

In addition, we conducted a post hoc analysis to examine whether explicit versus implicit learning of talker–reward associations differentially affected speech intelligibility. To this end, we constructed a new generalized linear mixed-effects model that included the between-subjects factor of exposure type (Experiment 1 vs. Experiment 2) in addition to the three predictors used in the main analysis. This model revealed no significant main effect of exposure type (*p* = 0.33) and no significant two-way interactions with TMR, target-talker value, or masker-talker value (all *p* values > 0.31). However, a significant three-way interaction between exposure type × TMR × target-talker value was observed, χ^2^(3) = 8.66, *p* = 0.034, but no other three-way or four-way interactions related to the exposure type (*p* = 0.64).

To further examine the significant three-way interaction with the type of talker-value exposures, we conducted follow-up analyses by fitting a separate reduced generalized linear mixed-effects model for each TMR level, including the within-subjects conditions of the target-talker and masker-talker values (high vs. low rewards) and the factor of exposure type. For the three higher levels of TMR (−15, −10, and −5 dB), there were no significant effects related to the type of talker-value exposures (all *p* values ≥ 0.14). However, in the −20 dB TMR condition, we found a significant interaction effect of the type of exposure × target-talker value, χ^2^(1) = 10.32, *p* = 0.0013; this effect confirms that the extent of speech intelligibility benefits from high-valued versus low-valued target talkers is significantly greater following incidental learning of talker–reward associations than following explicit learning of the same associations.

### Discussion

Experiment 2 results revealed that incidental exposure of talker–reward contingencies did not have any significant impact on listeners’ ability to ignore a distractor talker. Specifically, listeners’ speech processing was not interfered more when they had to ignore a high-valued distractor talker compared with low-valued talker. However, talker–reward training facilitated listeners’ ability to comprehend target talker’s speech. In the most challenging listening condition (−20 dB TMR), there was a speech intelligibility benefit when high-value talkers spoke the target sentence compared with those associated with low-value rewards. This benefit was observed despite listeners were not consciously aware of the talker–reward association. This finding might suggest that even though the reward-associated feature was not relevant to task goals, experiencing the talkers’ voices during reward learning could have enhanced development of talker-specific representations of speech; this in turn, facilitated listeners’ ability to attend and comprehend the target talker’s speech under highly challenging listening conditions. Furthermore, compared with Experiment 1, the blocked presentation of each talker during reward training might have encouraged listeners to better learn talker-specific acoustic–phonetic characteristics that are more relevant for speech intelligibility.

However, if talker–reward contingencies indeed enhance talker-specific representations that facilitate speech comprehension, this benefit should become more pronounced in situations where listeners must rely more heavily on talker-specific acoustic cues to segregate competing speech streams. In Experiments 1 and 2, spatial separation between the target and masker speech provided a strong cue for stream segregation (Arbogast & Kidd, 2000; Brungart, 2001; Durlach et al., 2003; Freyman et al., 1999, 2001; Kidd et al., 1998). Therefore, it is possible that spatial cues reduced listeners’ reliance on talker-specific features, thereby limiting the potential impact of reward-based learning. To test this possibility, we conducted a follow-up experiment in which listeners were required to comprehend speech while a same-gender masker stream originated from the same spatial location as the target talker. This manipulation was expected to increase information masking relative to the spatially separated streams used in the previous experiments.

## Experiment 3

The aim of this experiment was to examine whether the talker–reward contingency effect observed in Experiment 2 can be generalized to a more challenging listening condition. Specifically, if target speech is indeed more intelligible when spoken by a high-valued talker under adverse listening settings, then this effect should be stronger when spatial cues for stream segregation are removed. To test this possibility, listeners heard two competing talkers’ speech originating from the same location (co-located sources). Presenting the same gender voices without any spatial separation substantially increases masking compared with the spatially separated streams, thereby reducing speech intelligibility (Kidd et al., 2016; Oh et al., 2021). To avoid floor effects under this condition, we adjusted the four TMR levels by +10 dB TMR relative to those used in Experiments 1 and 2. The resulting TMR range was comparable to previous studies assessing talker familiarity benefit under co-located speech-on-speech masking (e.g., Domingo et al., 2020; Holmes et al., 2021; Johnsrude et al., 2013).

### Methods

#### Participants

A new sample of 30 Binghamton University undergraduates (20 women, 10 men; mean age: 20.1 ± 3.70, 18–37 years) participated this experiment and received either course credit or $15 per hour. All participants met the same inclusionary/exclusionary criteria, and all provided written informed consent.

#### Stimuli, task design, and procedure

The sound stimuli, the auditory 2-back to expose the talker–reward contingencies, and study procedures were identical to Experiment 2. Upon completing the exposure phase on the second day, participants were given the speech intelligibility test.

The test phase was modified to present the speech streams of the two talkers (target and masker) from the same spatial location at the center along the azimuth (i.e., no interaural time difference). Because presenting same-gender voices without spatial separation produces substantially stronger masking—typically reducing intelligibility by approximately 7–10 dB relative to spatially separated conditions (Kidd et al., 2016; Oh et al., 2021)—we adjusted the target-to-masker ratio (TMR) levels to avoid floor effects. Specifically, TMR levels ranged from −10 to +5 dB in 5 dB steps. These values are comparable to those used in previous studies employing similar speech-on-speech paradigms (e.g., Holmes et al., 2018; Johnsrude et al., 2013).

With the change in TMRs, we also modified the speech intelligibility test to direct listeners’ attention to target speech by cuing participants using the first word of the sentence. Specifically, participants were instructed to listen to the target sentence that began with a specific name (either “Bob” or “Pat”) and report the remaining four words from the that sentence. The target sentence name word was displayed on the screen while listening to the sentences, and remained constant through the test phase. The target sentence names were counterbalanced across participants. Participant’s target word identification score per trial were computed based on the total of four responses (cf. five responses in Experiments 1 & 2).

The total number of test trials, distribution of the trials across the conditions, and test procedure were identical to Experiments 1 and 2.

### Results

#### Results of exposure phase

**Fig. 8:**
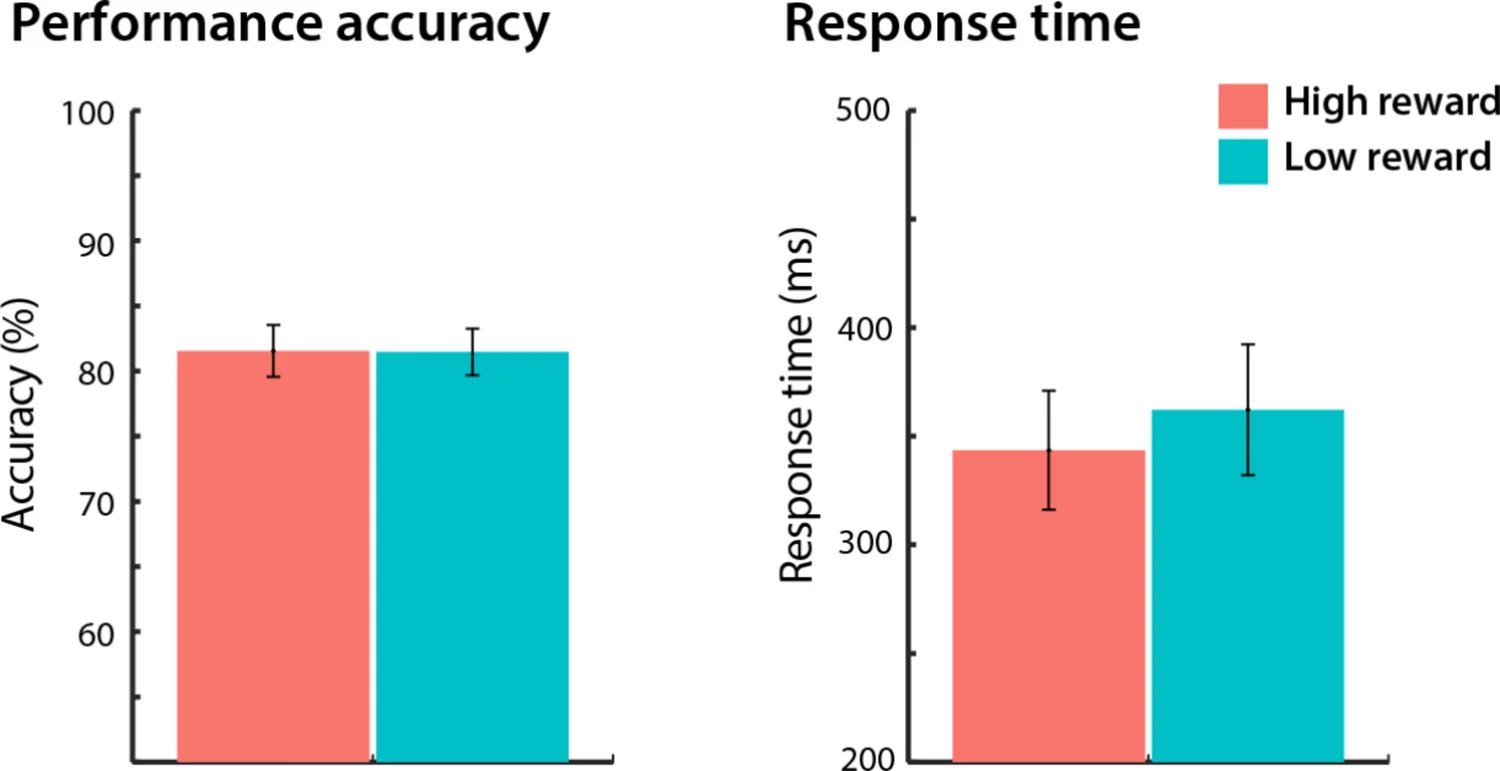
Average task performances in the auditory 2-back task (Experiment 3 exposure phase) in each talker–reward condition. Left, response accuracy. Right, average response time on the correct trials. The same illustration scheme as in Fig. 3 is used. (Color figure online)

Figure 8 and Table 6 show the overall performance during the auditory 2-back task for each talker–reward condition. The mixed-effects model on listeners’ auditory 2-back task accuracy did not reveal a significant effect of talker–reward value condition, χ^2^(1) = 1.96; *p* = 0.16. Similarly, the model analyzing response times (of correct responses) did not reveal a significant effect of talker–reward value condition, χ^2^(1) = 3.68; *p* = 0.055. These results are similar to the patterns found in Experiment 2, suggesting that listeners’ responses were orthogonal to the reward manipulation.

**Table 6:** Mean (± *SD*) accuracy and response time on performing the auditory 2-back task for each talker–reward condition in Experiment 3

	Accuracy (%)	Response time (ms)
High reward	81.55 ± 10.91	343.5 ± 150.0
Low reward	81.46 ± 9.77	362.1 ± 164.7

#### Results of test phase in correct identification of target speech

**Fig. 9:**
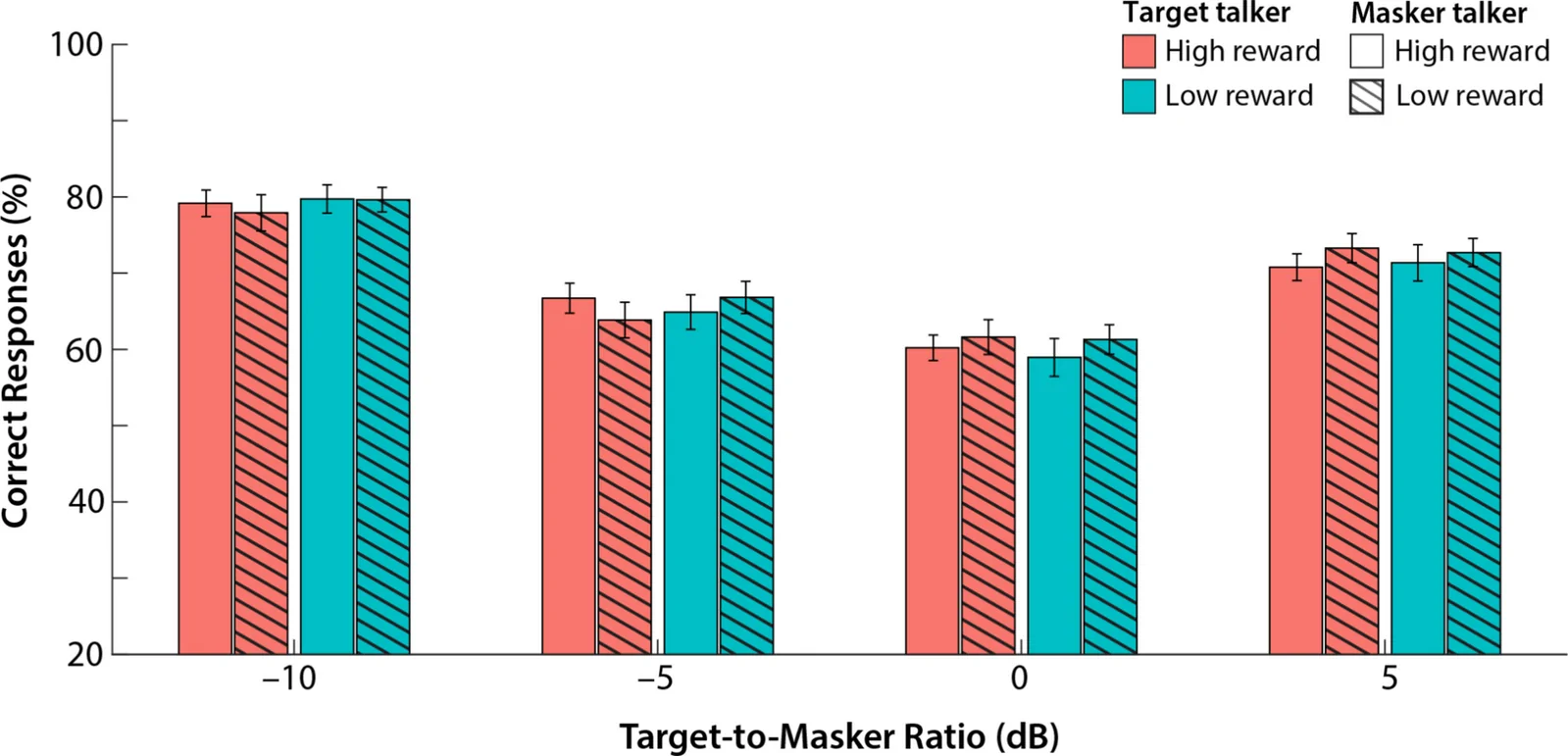
Speech intelligibility test accuracy of Experiment 3 for each condition varied in reward values of the target × masker talkers across the four TMR levels. (Color figure online)

Figure 9 illustrates the overall accuracy of target word identification across the four levels of TMR, in which the target and masker speech streams were presented without spatial separation. The generalized mixed-effects model on listeners’ accuracy in identifying target words revealed a significant main effect of TMR, χ^2^(3) = 229.69, *p* < 0.0001, but no significant main or interaction effects related to the talker–reward contingency associated with either the target or masker talkers (all χ^2^ values < 6.18, *p* values ≥ 0.10; Table 7).

**Table 7:** Mixed-effects logistic modeling results on the speech intelligibility test performance in Experiment 3

	χ^2^	*df*	*p*
TMR	229.64	3	< 0.0001*
Target talker value	0.48	1	0.49
Masker talker value	0.14	1	0.72
TMR × target talker value	6.18	3	0.10
TMR × masker talker value	2.00	3	0.57
Target talker × masker talker value	1.81	1	0.18
TMR × target talker × masker talker value	3.92	3	0.27

*TMR* target-to-masker ratio. * denotes significant effects Type III Wald χ^2^ test

Unlike Experiments 1 and 2, in which participants’ performance scaled linearly with the relative intensity of the target vs. masker speech stream, we found a U-shaped pattern of performance along the varying levels of TMR. As illustrated in Fig. 9, participants’ performance accuracy did not scale with increasing TMR levels. Instead, participants’ performance was lowest when the target and masker speech streams were presented at the same intensity (0 dB TMR), and the highest accuracy at the lowest TMR level (−10 dB), in which the intensity difference between the target and masker streams was greatest.

#### Results of test phase in masker confusion

We also examined the effects of the conditions on the masker confusion error rate (i.e., reporting words from the masker talker) using a linear mixed-effects model. We found a significant main effect of TMR, χ^2^(3) = 221.47, *p* < 0.0001, but the model did not reveal any significant effects related to the reward values of the target or the masker talkers (all χ^2^ values < 3.09; *p* values > 0.46).

The significant effect of TMR on masker confusion errors followed a similar, U-shaped trend of the performance accuracy across the TMR levels. Rather than the masker error systematically decreased with higher relative amplitude of the target talkers’ speech, confusion errors were highest when the target and masker speech streams were presented at the same intensity (0 dB TMR) and lowest in the condition with the largest intensity difference between the streams (−10 dB TMR; average masker error rate; *M*_–10 dB_ = 3.13 ± 0.08%, *M*_–5 dB_ = 6.05 ± 1.15%, *M*_0 dB_ = 7.26 ± 1.07%, *M*_+5 dB_ = 5.03 ± 1.13%).

### Discussion

By removing spatial separation of the speech streams, Experiment 3 was designed to create a more adverse listening condition than Experiments 1 and 2 in order to test the robustness of the target speech intelligibility enhancement from high versus low value-associated voices found in Experiment 2. We observed a robust effect of TMR on listeners’ speech intelligibility; however, we did not observe a reliable effect of talker–reward contingencies here regardless of the value associated with to-be-attended target or to-be-ignored masker talkers. We predicted that if high-valued voices enhance speech intelligibility especially under adverse listening conditions, we expected that the reward effect to be even stronger in Experiment 3. However, no such enhancement was observed.

The effect of TMR also followed an interesting pattern. Unlike Experiments 1 and 2, we observed that performance accuracy was affected by TMR nonlinearly, such that listeners achieved the highest accuracy at the lowest TMR level (−10 dB) and the lowest performance when the target and masker speech streams were presented at the same intensity level (0 dB TMR). This pattern suggests that the difficulty of the listening condition was not determined solely by the absolute intensity of the target speech relative to the masker. Instead, the perceptual similarity between the two streams, particularly when they were presented at comparable intensity levels, appears to have played a more important role in determining speech intelligibility (e.g., Ihlefeld & Shinn-Cunningham, 2008).

## General discussion

The goal of the present study is to examine the value-driven attentional effects within this context of speech processing in multitalker listening environments. Specifically, we tested whether listeners’ comprehension of speech depends on the learned behavioral value/importance of the talker. By utilizing reward-based learning paradigms to associate talkers’ voices with different magnitudes of reward, we examined whether prior reward associations with talkers’ voices influence listeners’ ability to understand target speech in the presence of a competing talker.

Across three experiments, we found little consistent evidence that talker–reward contingencies influenced listeners’ ability to identify target speech. In Experiment 1, explicit talker–reward exposure through a voice identification task did not enhance intelligibility when high-value talkers served as targets, nor did it increase interference when high-value talkers served as maskers. In Experiment 2, incidental exposure to talker–reward relationships produced a modest intelligibility advantage for high-reward talkers, but this effect emerged only under the most adverse listening condition. However, this effect did not replicate in Experiment 3, when we imposed greater perceptual demands—higher information masking—by removing spatial separation between the two same-gender talkers.

Rather than reward history linked to talkers’ voices, we found that speech intelligibility was robustly affected by acoustic factors, particularly the target-to-masker ratio (TMR). These findings suggest that talker–reward associations do not reliably modulate listeners’ ability to parse speech in the presence of competing streams. Instead, listeners appear to rely primarily on available acoustic cues that facilitate stream segregation and selective attention. Taken together, the present findings provide limited support for the hypothesis that value-driven attentional biases generalize to complex auditory objects such as talkers’ voices during multitalker speech perception.

### Value-driven attention in the context of talker voices

Solving the cocktail problem (Cherry, 1953) hinges on one’s ability to segregate sound sources (Bregman, 1990) and attend to a target stream while filtering out other irrelevant sounds (Fritz et al., 2007; Shinn-Cunningham, 2008; Sussman, 2017). Because processing resources are limited, there is biased competition between volitional goals and physical salience to determine which object gains attentional focus (Desimone & Duncan, 1995; Shinn-Cunningham, 2008). According to the value-driven attention framework, one potential factor that may influence competition for limited attentional resources is the reward history associated with sensory objects (Awh et al., 2012). A large body of research in vision has demonstrated that reward associations can bias attentional allocation, leading observers to preferentially process stimuli that were previously associated with reward (Chelazzi et al., 2013; Kiss et al., 2009; Krebs et al., 2010; Kristjansson et al., 2010; Serences, 2008). Within this framework, reward-associated stimuli would gain priority in attentional competition (Della Libera & Chelazzi, 2009; Maunsell, 2004).

Applying this framework to speech processing under competition with other concurrent speech, we predicted that talkers’ voices would serve as distinct auditory objects, which would gain enhanced salience when paired with high rewards. Given that the benefit of talker familiarity on speech intelligibility found to be most notable in cocktail party situations, such as yielding robust talker-specific speech representations against other distractor talkers’ speech (Holmes & Johnsrude, 2020, 2021), we expected that effects of talker–value association to be manifested in a similar manner. Specifically, we expected that speech from high-reward talkers would be more intelligible when they serve as targets, and high-reward talkers would increase interference when they serve as maskers. However, the present results suggest that reward-based prioritization does not robustly extend to processing multitalker speech streams. Even when listeners were explicitly trained to associate different talkers with high versus low rewards (Experiment 1), reward history did not reliably influence speech intelligibility. This finding indicates that associating differential reward values with talker identities is insufficient to affect auditory object formation and to alter auditory object selection under spatially segregated listening conditions.

Experiment 2 provided a limited indication that reward history might influence speech processing. While Experiment 2 listeners were exposed to talker–reward associations incidentally, talkers were presented in separate blocks during training, potentially allowing listeners to better extract each talker’s acoustic–phonetic characteristics. After this training, listeners showed a small intelligibility advantage for high-reward talkers only at the most adverse listening condition (−20 dB TMR). This pattern appears to be in line with prior studies in vision that demonstrated the recognition bias towards high-rewarded objects when attentional resources are highly constrained (Chelazzi et al., 2013, 2014; Raymond & O’Brien, 2009).

However, this facilitatory effect appears to be fragile. In Experiment 3, we created an even more challenging listening condition compared with those of Experiments 1 and 2; by removing spatial separation between the two speech streams, listeners were required to rely more heavily on talker-specific acoustic–phonetic characteristics to segregate the two competing talkers. Under this demand, the reward-related target speech processing advantage disappeared. Overall, these findings suggest that reward-related modulation associated with talkers’ voices is weak, and it does not reliably overcome the perceptual demands imposed by informational masking.

Similarly, reward values of masker talkers did not impact processing of target speech. Across three experiments, the values attributed to the masker talkers had no discernible impact; there was neither disruption nor enhancement in parsing target speech when the masker talker was associated with high versus low-valued rewards. This finding contrasts with the effects typically associated with value-driven attentional capture; that is, reward influences attentional control, causing goal-directed attention to be automatically diverted to stimuli previously associated with high rewards, irrespective of their physical salience or task relevance (e.g., Anderson & Yantis, 2013; Anderson et al., 2011a, 2011b; MacLean & Giesbrecht, 2015; MacLean et al., 2016).

One possible explanation for the absence of reward effects is insufficient amount of training or the use of probabilistic reward distributions leading to relatively weak reward contingencies. However, this explanation seems unlikely. Both of these factors in the current study (i.e., the number of trials during reward training and 80/20% reward probabilities) were based on the prior work by Anderson et al. (2011a, 2011b) that observed reliable value-driven attentional capture. Moreover, our training was given across 2 days, which should have enhanced reward-based perceptual learning through sleep-based memory consolidation (Tamaki et al., 2020). Also, given how even a short-term experience with talkers’ voices (as little as 10 min) can yield talker familiarity advantage in speech intelligibility (Holmes et al., 2021), the amount of exposure to talker–reward contingencies is unlikely to account for the lack of reward effects.

Another possibility is that the complexity of the features associated with the rewards may account for the absence of value-driven attentional capture towards the masker speech. Studies that show reliable value-driven attentional capture have typically linked reward values to simple perceptual features, like two easily distinguishable colored singletons in visual search tasks (Anderson et al., 2011a; Anderson & Yantis, 2013; Hickey et al., 2010). Similarly, a few existing auditory studies showing value-driven attentional capture utilized two highly distinct tones associated with positive rewards (Asutay & Västfjäll, 2016) or highly distinct words (i.e., specific spoken letters in Anderson, 2016b; Kim et al., 2021). Consistent with this interpretation, prior research has failed to capture attention when reward values were associated with nonsalient (Milner et al., 2023; Wang et al., 2013) or complex conjunctions of features (e.g., faces, ambiguous shapes, words; Della Libera & Chelazzi, 2006, 2009; Raymond & O’Brien, 2009; Rutherford et al., 2010). Therefore, it is plausible that associating rewards with talkers’ voices, which inherently are composite of multiple acoustic features, whereas the words are highly variable, may not be efficiently processed to capture attention involuntarily.

More importantly, evidence for reward-based modulation of complex speech processing has been inconsistent. Prior speech studies have largely observed reward effects at a post-perceptual decision level rather than at a perceptual locus (e.g., Connine & Clifton, 1987). In fact, the absence of robust reward effects closely aligns with a recent study by Mechtenberg et al. (2025) that trained listeners to associate varying rewards with distinct talkers’ voices. Although the testing context was centered to examine the effect of talker–reward exposure on the extent of listeners’ talker-specific phonetic calibrations, the reward effect on talker-specific perceptual learning was not observed in Mechtenberg et al. (2025). The present findings extend this pattern by showing that reward history does not reliably modulate speech intelligibility under informational masking. Together, these results suggest that value-driven attentional biases observed for simple or discrete auditory stimuli may not readily scale to complex, socially meaningful auditory objects such as talkers’ voices.

### The dominant cues in speech-on-speech competition

Across all experiments, listeners’ performance was strongly influenced by acoustic cues that support stream segregation. In Experiments 1 and 2 where listeners heard two spatially separated speech streams, target-to-masker ratio (TMR) exerted a robust effect on intelligibility: performance declined linearly as TMR decreased. Despite the listening challenge due to low intensity of target speech, listeners achieved relatively good accuracy even at −20 dB TMR, likely by relying on spatial separation between the two competing speech streams. Because spatial location serves as a robust cue for guiding top-down, volitional auditory attention (e.g., Arbogast & Kidd, 2000; Broadbent, 1954; Mondor & Zatorre, 1995), spatial separation itself, even though listeners heard two talkers of same gender, could effectively aid in ignoring the irrelevant masker stream due to spatial release from masking (Freyman et al., 1999, 2001; Jones & Litovsky, 2008; Litovsky, 2012; Kidd et al., 1998, 2016; Oh et al., 2021).

When spatial cues were removed in Experiment 3, listeners’ performance appeared to depend more strongly on intensity differences between the two streams rather than on the talkers’ voices. Interestingly, listeners’ performance accuracy did not monotonically increase with TMR. Instead, performance was lowest when the two streams were presented at the same intensity (i.e., 0 dB TMR), whereas at −10 dB TMR, arguably the most challenging condition, listeners were able to comprehend the target speech significantly better than other higher TMR levels, including +5 dB TMR. This non-monotonic pattern suggests that perceptual similarity between the streams made segregation more difficult, particularly when hearing two same-gender talkers.

This finding is highly consistent with previous studies demonstrating that listeners prioritize reliable physical cues, including the differences in intensity, when organizing auditory scenes (e.g., Brungart, 2001; Ihlefeld & Shinn-Cunningham, 2008). Thus, it is an open question for future research to examine whether systematically manipulating acoustic distinctiveness between talkers might allow learned salience, such as reward associations, to play a stronger role in guiding stream segregation and selective attention.

### Conclusion

The present findings suggest that value-driven attentional mechanisms may have more limited generalizability to complex speech processing than previously assumed. Across three experiments, associating rewards with talkers’ voices did not reliably enhance speech intelligibility or increase interference from competing speech. Instead, listeners consistently relied on acoustic cues, such as spatial separation and target-to-masker ratio, that directly support auditory scene segregation. These results indicate that learned value associated with a talker’s voice does not readily override the acoustic constraints imposed in the context of speech-on-speech perception.

Although reward learning can bias attention toward simple stimuli, the present findings indicate that such value-driven biases may not scale to complex auditory objects defined by multidimensional and dynamically varying features, such as talkers’ voices. By directly testing reward generalization within a speech-on-speech paradigm, this study provides an important insight into understanding the limitation of value-driven attention in the auditory domain.

## Data Availability

The data and materials are available online (https://osf.io/4gu6s/). The experiments were not preregistered.

## Code availability

The analysis script is available online (https://osf.io/4gu6s/).

## Declarations

### Open practices statement

The trial-level data for all experiments and voice stimuli are available at https://osf.io/4gu6s/. The experiments were not preregistered.
